# Sulfadiazine plasma concentrations in women with pregnancy-acquired compared to ocular toxoplasmosis under pyrimethamine and sulfadiazine therapy: a case–control study

**DOI:** 10.1186/s40001-020-00458-7

**Published:** 2020-11-23

**Authors:** Ingrid Reiter-Owona, Harald Hlobil, Martin Enders, Ute Klarmann-Schulz, Barbara Gruetzmacher, Veronika Rilling, Achim Hoerauf, Justus G. Garweg

**Affiliations:** 1 Institute of Medical Microbiology, Immunology and Parasitology, University Hospital of Bonn, Bonn, Germany; 2 Laborärzte Sindelfingen, Sindelfingen, Germany; 3 Labor Prof. Gisela Enders Und Kollegen, Stuttgart, Germany; 4 Institute for Medical Biometry, Informatics and Epidemiology, University Hospital of Bonn, Bonn, Germany; 5 Deutsches Zentrum Für Infektionsforschung (DZIF) E. V., Braunschweig, Germany; 6 Swiss Eye Institute, Berner Augenklinik am Lindenhofspital, Bremgartenstrasse 119, CH-3012 Bern, Switzerland; 7 Department of Ophthalmology, Inselspital, University of Bern, Bern, Switzerland

**Keywords:** Primary toxoplasmosis, Ocular toxoplasmosis, Pyrimethamine, Sulfadiazine, Plasma concentration, Liquid chromatography–mass spectrometry

## Abstract

**Background:**

Dosing recommendations for the treatment of pregnancy-acquired toxoplasmosis are empirical and widely based on experimental data. There are no pharmacological data on pregnant women with acute *Toxoplasma gondii* infection under treatment with pyrimethamine (PY) and sulfadiazine (SA) and our study intends to tighten this gap.

**Methods:**

In this retrospective case–control study, we included 89 pregnant women with primary *Toxoplasma* infection (PT) treated with PY (50 mg first dose, then 25 mg/day), SA (50 mg/kg of body weight/day), and folinic acid (10–15 mg per week). These were compared to a group of 17 women with acute ocular toxoplasmosis (OT) treated with an initial PY dose of 75 mg, thereafter 25 mg twice a day but on the same SA and folinic acid regimen. The exact interval between drug intake and blood sampling and co-medication had not been recorded. Plasma levels of PY and SA were determined 14 ± 4 days after treatment initiation using liquid chromatography–mass spectrometry and compared using the Mann–Whitney *U* test at a *p* < 0.05 level.

**Results:**

In 23 PT patients (26%), SA levels were below 20 mg/l. Fifteen of these 23 patients (17% of all patients) in parallel presented with PY levels below 700 µg/l. Both drug concentrations differed remarkably between individuals and groups (PY: PT median 810 µg/l, 95% CI for the median [745; 917] vs. OT 1230 µg/l [780; 1890], *p* = 0.006; SA: PT 46.2 mg/l [39.9; 54.4] vs. OT 70.4 mg/l [52.4; 89], *p* = 0.015) despite an identical SA dosing scheme.

**Conclusions:**

SA plasma concentrations were found in the median 34% lower in pregnant women with PT compared to OT patients and fell below a lower reference value of 50 mg/l in a substantial portion of PT patients. The interindividual variability of plasma concentrations in combination with systematically lower drug levels and possibly a lower compliance in pregnant women may thus account for a still not yet supportable transmission risk. Systematic drug-level testing in PT under PY/SA treatment deserves to be considered.

## Background

Maternal infection with *Toxoplasma gondii* during pregnancy may lead to transmission of the infection to the foetus. Children with congenital *Toxoplasma* infection may be completely asymptomatic (with subclinical infection) or develop severe clinical symptoms, such as hydrocephalus, retinochoroiditis, or intracranial calcifications. In children with subclinical infection, the parasite can re-activate later in life and induce retinochoroiditis (ocular toxoplasmosis [OT]). To reduce the risk of transmission and congenital toxoplasmosis, early treatment of newly infected pregnant women is justified [1–5]. A combination of sulfadiazine (SA) and pyrimethamine (PY) is considered most effective, as both drugs act synergistically, pass the placenta, and accumulate in the maternal and foetal tissues. Observational studies have demonstrated an association of prenatal treatment with the prevention of symptomatic disease in infants [6].

The parasitostatic effect of SA and the parasitocidal effect of PY, as well as the initial dosing strategies, were first described in the late 1950s [7–9], and the in vitro activities of both drugs were confirmed later for different strains of *T. gondii* [10, 11]. Studies on experimentally infected animals, in vitro studies, and studies with immunosuppressed individuals have confirmed the efficiency of this drug combination in blocking the parasite’s replication process [7, 12–15]. Nevertheless, it has been difficult to prove the efficacy of these drugs in immunocompetent individuals and foetuses. Studies on rhesus monkeys indicate that if administered soon after infection, the drug combination can reduce the parasite load in foetal tissue to undetectable levels [13]. In human congenital toxoplasmosis, it is still not clear whether treatment failures are due to late treatment onset after maternal infection or to ineffective drug concentrations in the foetal tissue [16]. In vitro studies have demonstrated that the drugs act in a concentration-dependent fashion. When used in combination, the plasma concentrations in mice should reach at least 100 µg/l for PY and 25 mg/l for SA [17]. In rhesus monkeys, maximum concentrations of 220 µg/l for PY and 58.7 mg/l for SA were reached with a drug regimen that was also applied to humans [13].

Therapeutic drug monitoring in *Toxoplasma*-infected patients has revealed that plasma concentrations not only vary between patients and different patient groups, but they are also unpredictable, even under standardized therapy [2, 18–21]. So far, plasma concentrations within a range of 700–1300 µg/l (PY) and 50–150 mg/l (SA) may be assumed effective in humans [14, 22]. Folinic acid has to be administered concomitantly to prevent bone marrow suppression, which as a toxic side effect of PY. Data on the pharmacokinetics of PY and SA exist predominately for HIV-positive males [15] and children with congenital toxoplasmosis [2, 18, 19, 21, 23]. Nevertheless, there are still no pharmacological data from pregnant women with acute *Toxoplasma* infection under treatment with PY and SA [18]. The unsatisfying efficacy of the combination treatment to prevent vertical transmission still deserves to be explained on pharmacological grounds. We thought that comparing plasma concentrations of PY and SA in pregnant women with pregnancy-acquired toxoplasmosis to those in females with OT might help to understand the role of pregnancy-associated pharmacological factors. Based on similar patient characteristics and a comparable treatment protocol, our case–control study aimed to identify possible differences in PY and SA plasma concentrations in pregnant and non-pregnant women.

## Methods

### Patients

This retrospective case–control study covers the period from 1997 until 2011, during which plasma samples had been submitted for drug-level testing from a consecutive series of 89 pregnant women aged 18.8 to 43.8 years (mean 29.6 ± 6.0, [95% confidence interval: 28.4; 30.9]) receiving anti-parasitic treatment for proven or suspected primary *Toxoplasma* infection (PT) (Table 1). All women had reached or passed the 16^th^ week of pregnancy when they received the combination therapy according to the following standard dosages: PY 50 mg on the first day, then 25 mg/day and SA 50 mg/kg of body weight/d up to a maximum dosage of 4.0 g/day, divided into three to four doses per day that were supplemented with folinic acid (10–15 mg per week) according to the recommendation of the German federal health authorities, Robert Koch Institute protocol established in 1988 (https://www.rki.de/DE/Content/Infekt/EpidBull/Merkblaetter/Ratgeber_Toxoplasmose.html). Drug prescription and patient supervision were effected by their respective obstetricians. Treatment lasted a minimum of 4 weeks and began right after the diagnosis of *Toxoplasma* infection had been confirmed. Blood or plasma samples were collected approximately 14 days after treatment onset and sent to the Southern German reference laboratory (Laboratory Harald Hlobil, Sindelfingen, Germany) to quantify the plasma concentrations of PY and SA.

**Table 1:** Baseline characteristics and plasma drug concentrations in pregnant women with pregnancy-associated primary toxoplasmosis and non-pregnant females with acute ocular toxoplasmosis, both assayed in the advisory laboratory using liquid chromatography–mass spectrometry (LC–MS)

	OT	PT	*p-*value
Age			
*N*	17	89	*p* = 0.024^a^
Mean ± SD [95% CI]	26.1 ± 5.3 [23.6; 28.5]	29.6 ± 6.0 [28.4; 30.9]	
Min–max	17–35	18.8–43.8	
Median	27	29.9	
Percentiles (25th; 75th)	21; 29	24.2; 33.6	
PY (µg/l)			
*N*	17	89	*p* = 0.006^b^
Mean ± SD	1550 ± 1411	838 ± 434	
Min–max	29–6160	29–2550	
Median [95% CI]^d^	1230 [780; 1890]	810 [745; 917]	
Percentiles (25th; 75th)	780; 1980	622; 1001	
SA (mg/l)^c^			
*N*	17	89	*p* = 0.015^b^
Mean ± SD	69.1 ± 44.2	44.5 ± 25.9	
Min–max	9.0–166.0	9.0–137.0	
Median [95% CI]^d^	70.4 [52.4; 89]	46.2 [39.9; 54.4]	
Percentiles (25th; 75th)	52.4; 89.0	21.8; 62.5	

^a^ *t*-test for independent samples

^b^ Mann–Whitney *U* test

^c^ Detection limit set to 9.0 mg/l

^d^ 95% confidence intervals of the median were calculated using bootstrapping

Serum samples from 17 HIV-negative women of similar age (17–35, mean 26.1 ± 5.3 [23.6; 28.5] years) who had been treated at the Uveitis Clinic of the University Hospital Bern (Inselspital) for acute symptomatic *Toxoplasma* retinochoroiditis between 1992 and 2001 were available for comparison (Tables 1 and 2). These patients with OT had initiated treatment with a loading dose of 75 mg PY, followed by 25 mg PY given twice daily, whereas the same SA dosage as in the PT group was given for a minimum of 6 weeks. Blood samples were routinely drawn from all individuals for side effect control approximately 14 days after treatment initiation (range 11–17 days). Unused samples were stored at − 18 °C in a biobank until their analysis in 2011. To confirm the stability of PY and SA in plasma during long-term storage at − 18 °C, we additionally included samples from 10 HIV-negative male patients treated during the same period for acute OT for which the same sampling, storage, and analysis protocols had been followed. Baseline characteristics of this group are displayed in Table 2. More recent blood samples from patients treated after 2001 were not available, since the treatment protocol had been changed from PY/SA to the fix combination pyrimethamine and sulfadoxine (Fansidar®) in 2001 and to the fixed-dose combination of trimethoprim 160 mg and sulfamethoxazole 800 mg twice a day by 2004. Since all individuals were outpatients, no information pertaining to the exact times of drug intake and blood sampling was available. As a result, it was impossible to calculate individual trough-to-peak ratios.

**Table 2:** Baseline characteristics and plasma drug concentrations in males and non-pregnant females with acute ocular toxoplasmosis, both assayed in the advisory laboratory using liquid chromatography–mass spectrometry (LC–MS)

	OT		*p-*value
	Male^a^	Female^b^	
Age			
*N*	10	17	*p* = 0.841^a^
Mean ± SD [95% CI]	26.6 ± 8.7 [20.4; 32.8]	26.1 ± 5.3 [23.6; 28.5]	
Min–max	16–48	17–35	
Median	25	27	
Percentiles (25th; 75th)	21; 29	21; 29	
Days after treatment start			
*N*	10	17	*p* = 0.083^b^
Mean ± SD	13 ± 2	14 ± 2	
Min–max	10–15	11–17	
Median [95% CI]^d^	14 [12; 14]	14 [14; 16]	
Percentiles (25th; 75th)	12; 14	13; 16	
PY (µg/l)			
* N*	10	17	*p* = 0.537^b^
Mean ± SD	1527 ± 667	1550 ± 1411	
Min–max	789–2630	29–6160	
Median [95% CI]^d^	1321 [962; 2140]	1230 [780; 1890]	
Percentiles (25th; 75th)	962; 2140	780; 1980	
SA [mg/l)^c^			
*N*	10	17	*p* = 0.386^b^
Mean ± SD	82.4 ± 47.9	69.1 ± 44.2	
Min–max	9.0–159.0	9.0–166.0	
Median [95% CI]^d^	82.4 [53.5; 115.0]	70.4 [52.4; 89]	
Percentiles (25th; 75th)	53.5; 115	52.4; 89.0	

^a^ t-test for independent samples

^b^ Mann–Whitney U test

^c^ Detection limit set to 9.0 mg/l

^d^ 95% confidence intervals of the median were calculated using bootstrapping

### Determination of plasma concentrations

Plasma concentrations of PY and SA were assayed in the same advisory laboratory using chromatography on the day of blood collection (PT) or after thawing of the stored samples (OT). Analysis was done by liquid chromatography–mass spectrometry (HPLC–MS/MS 3200 Q Trap, Sciex, Germany). For this, 50 μl of plasma/serum sample was mixed with 225 μl of methanol (MeOH; Rotisolv HPLC-Grade, Roth Germany), 25 μl of acetonitrile (Rotisolv pestilyse, Roth, Germany) and an internal standard (droperidol) as systematic test performance control and homogenized for 1 min, before the mixture was precipitated for 10 min at 13,000 rpm. In all, 100 µl of the supernatant was used for LC–MS according to the manufacturer’s instructions. Four additional specific internal standards (ISs) were prepared by spiking drug-free serum with known amounts of PY (Sigma Aldrich, Germany, P-7771) or SA (LGC Standards). The concentrations of the ISs for PY were level 1 (200 µg/l), level 2 (400 µg/l), level 3 (1000 µg/l), and level 4 (2000 µg/l) and for SA were level 1 (10 mg/l), level 2 (20 mg/l), level 3 (50 mg/l), and level 4 (100 mg/l). Low positive controls (PY 500 µg/l, SA 25 mg/l) and high positive controls (PY 1200 µg/l, SA 60 mg/l) were added. The described method (hereafter named LC–MS) was validated and proved to be sensitive, selective, and accurate for quantification of PY and SA in human serum/plasma samples. Intra-assay coefficients of variation (CVs) were as follows: for PY control 1 (500 µg/l): CV = 2.0%, and control 2 (1200 µg/l): CV = 0.9% and for SA control 1 (25 mg/l): CV = 2.10% and control 2 (60 mg/l): CV = 0.9%. The ISs were confirmed to be stable under storage at ≤ 16 °C for 1 year. No peaks interfering with quantification were observed throughout the validation process. The assay had lower detection limits of 30 µg/l for PY and 9 mg/l for SA. Calculated intra- and interday CVs remained below 10%.

### Statistics

SPSS (IBM SPSS Statistics for Windows, Version 22.0. Armonk, NY: IBM Corp.) and SAS 9.4. (SAS Institute Inc., Cary, NC, USA) were used to analyse the data. Parametric data (age, days after initiation of therapy) are given in the text with range, mean ± standard deviation (SD) and corresponding 95% confidence intervals (CIs). The non-parametric serum concentrations of PY and SA are shown in the text as medians and corresponding 95% CIs. The 95% CIs of the medians were calculated with SPSS using bootstrapping. Differences between OT and PT were analysed using the *t*-test for independent samples for age and days after initiation of treatment and the Mann–Whitney *U*-test for the serum concentration of PY and SA. A p-value < 0.05 was considered significant.

## Results

The baseline characteristics of both groups are displayed in Table 1.

**Fig. 1:**
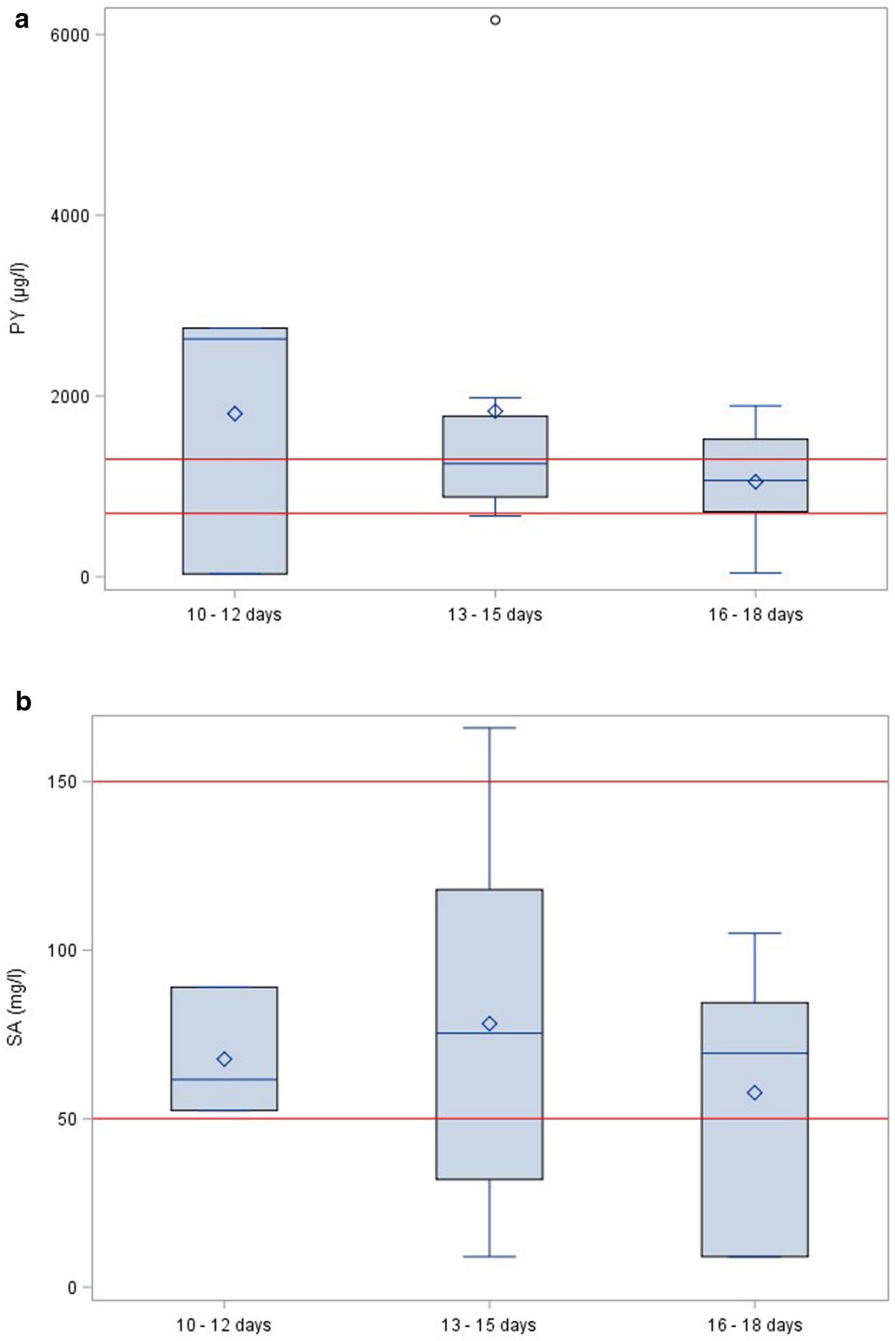
Boxplots of observed concentrations of pyrimethamine (**a**) and sulfadiazine (**b**) versus time during the early treatment phase in female patients with acute ocular toxoplasmosis (OT). Red lines: lower and upper target concentrations of pyrimethamine (700 and 1300 µg/l)^14^ and sulfadiazine (50 and 150 mg/l)^21^

By grouping OT samples according to the time of sampling (Group 1: 10–12 days, Group 2: 13–15 days, and Group 3: 16–18 days after treatment initiation), we observed no difference in plasma concentrations, indicating that both PY and SA concentrations had already reached a steady-state by the time of blood sampling (Fig. 1a, b).

**Fig. 2:**
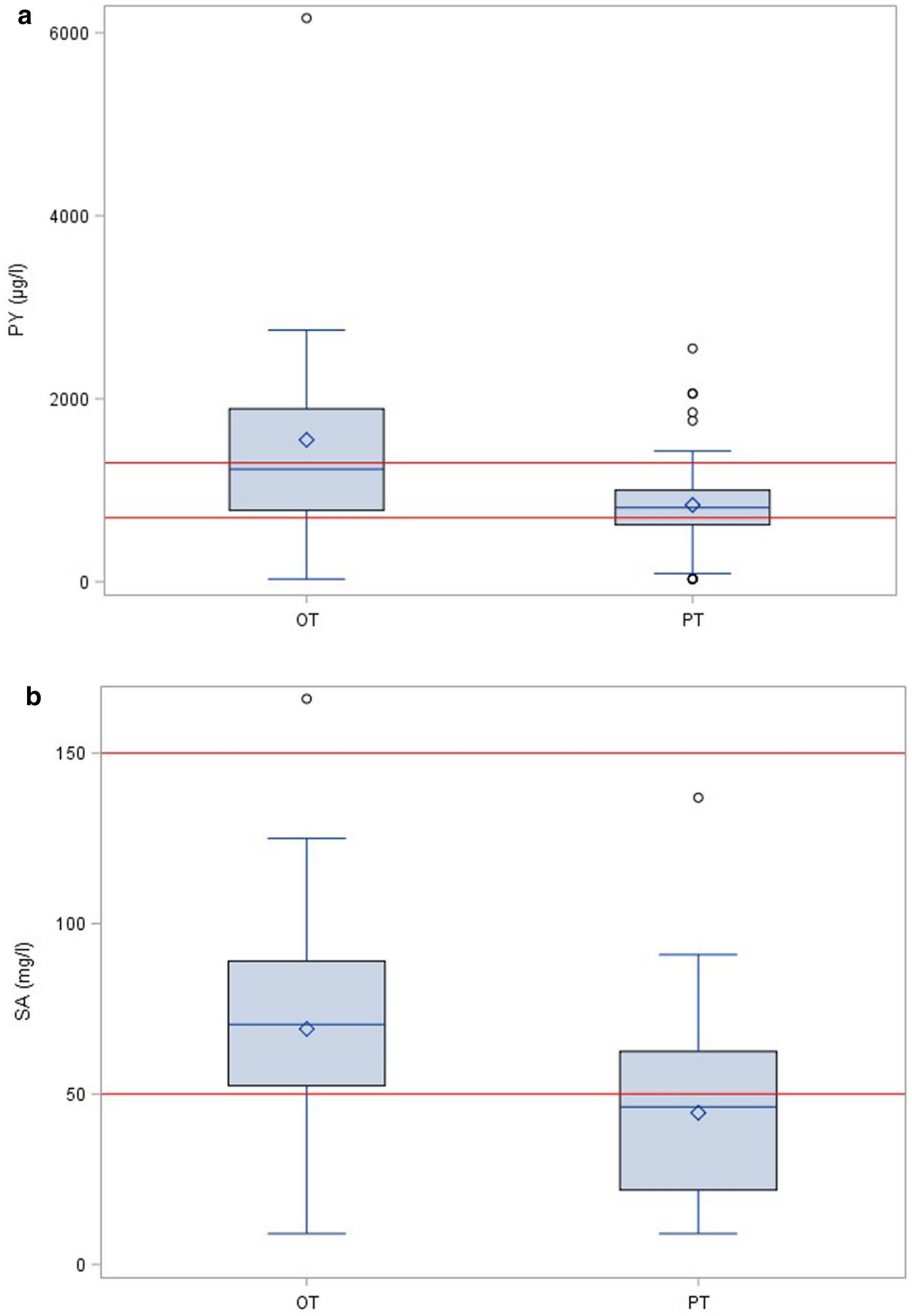
Comparison of the steady-state plasma concentrations of pyrimethamine (**a**) and sulfadiazine (**b**) in pregnant women (PT; *N* = 89) and in women with ocular toxoplasmosis (OT; *N* = 17). Red lines: lower and upper target concentrations of pyrimethamine (700 and 1300 µg/l)^14^ and sulfadiazine (50 and 150 mg/l)^21^

Based on the steady-state results in the OT group, we assumed that the PT group would also reach their steady-state plasma concentrations of PY and SA after this time. By comparing the median values of both drugs for both groups, we found that the PY levels (Fig. 2a) were 34% higher in women with OT (1230 [780; 1890] µg/l) compared to those in pregnant women with PT (810 [745; 917] µg/l; *p* = 0.006), which is in line with the difference in dosing (50 mg/day vs. 25 mg/day). The SA levels also differed by 34% (PT 46.2 [39.9; 54.4] mg/l vs. OT 70.4 [52.4; 89] mg/l; *p* = 0.015), despite an identical SA dosing scheme in both groups. If we assume, based on published evidence, an upper concentration limit for PY of 1700 µg/l and a lower concentration limit for SA of 50 mg/l, a majority of PT patients were, according to the reference values, underdosed for SA. The majority of OT samples, in contrast, showed PY concentrations above the target value (Fig. 2b). In 23 of the 89 PT patients (26%), SA levels dropped below 20 mg/l. Fifteen of these 23 patients (17% of all patients) in parallel presented with PY levels below a target concentration of 700 µg/l.

A comparison of the serum concentrations in female and male patients with acute OT under treatment with PY and SA revealed similar values for both drugs (Table 2). As the concentrations for males are in good agreement with published pharmacokinetic results after the immediate work-up of unfrozen plasma samples [23, 24], the different storage conditions for PT and OT samples cannot account for the observed differences in the plasma concentrations of either drug.

## Discussion

For SA, concentrations of 50–150 mg/l are considered therapeutic for most infections [22], whereas 26% of our PT patients presented with SA levels below 20 mg/l and 17% (15 patients) did not reach the targeted concentrations for both drugs, PY and SA. This is well in line with the clinically reported transmission rates in Europe of 5–13% [3, 25]. Despite an identical treatment regimen for SA, lower drug levels were observed in pregnant patients compared to those in non-pregnant women with OT: the median SA plasma concentration in PT patients was more than 34% (46.2 vs. 70.4 mg/l) lower than that in the OT group, indicating that not the treatment protocol per se is insufficient.

Physiological and metabolic changes during pregnancy may account for the lower SA concentrations. Maternal antibiotic concentrations have been found to be generally 10–50% lower compared to those in the non-pregnant state [26]. The pregnancy-related increase in total body fluid, a higher clearance rate or differences in body weight could affect the SA plasma concentrations [27, 28]. Not explained by physiological changes of metabolism and drug turnover in pregnancy [13] is the fact the two-thirds of patients with SA concentrations below the expected levels in parallel demonstrated significantly reduced PY levels (< 700 µg/l). A limited treatment compliance, a known phenomenon during pregnancy in other diseases [29, 30], may thus have contributed to these outcomes.

The clinically SA plasma concentrations found are comparable to those found in experimental toxoplasmosis in rhesus monkeys who reached peak concentrations of 58.7 mg/l [13]. This indicates that the suggested SA target levels (50–150 mg/l) [22] are not realistic or not needed in the presence of PY concentrations above 700 µg/l. SA is rapidly absorbed in the gut and eliminated mainly by acetylation with the urine. The elimination half-time of SA is 6–12 h in individuals with normal renal and hepatic function [24].

One obvious yet unavoidable limitation for our retrospective study was that the elapsed time between oral drug intake and blood sampling had not been recorded at the outpatient clinics. The elimination half-life for SA in monkeys was found to be about 5 h, implying an estimated deviation of measured to peak values of 50% [13]. This was taken into account in our series by a robust lowering of the cut-off setting for SA to 20 mg/l. An estimated deviation of 30% between trough and peak concentrations has to be balanced against a relatively large sample size (89 patients with PT) in this series. But, we have to admit that data about possible co-medications and their impact on drug levels are not available. The obviously large interindividual variation in plasma concentrations (by a factor of five), however, may not fully be explained by variation in the time lapse between intake of drug and blood sampling and co-medication [13]. Moreover, the significant inter-group difference in mean plasma concentrations cannot readily be explained with differences in the time gap between drug intake and blood sampling.

A further major limitation of our study is its retrospective design, which may be outweighed by a relatively large patient sample size. The determination of plasma concentrations in women beyond the 16th week of gestation was triggered by suspected or proven *Toxoplasma* seroconversion during pregnancy and performed as a clinical routine analysis over several years. The resulting lack of more specific information about body size, weight, general health and comorbidities and their impact in pregnant patients limits the interpretation of single patient results, whereas we think that the tendency in the large patient group of pregnant women is robust. The sample size of the second group of patients with OT was remarkably smaller, but we had access to the clinical data of these patients, which showed an age range that compares well to the pregnant women. None of these patients had significant comorbidities or a corresponding treatment, and in no case, an underlying renal or hepatic disease was documented. Increasing the sample size of the second group was not possible due to a change in the treatment protocol for OT after 2001 as outlined above.

The plasma concentrations were determined from blood samples collected approximately 14 d into treatment, assuming that both drugs would have reached a steady state by then, an assumption that was based on a subgroup analysis in non-pregnant (OT) women (Fig. 1a, b). Still, considering that pregnant women are usually excluded from pharmacokinetic studies due to ethical concerns, our study is one of the few available in this area of research. Dosing of anti-parasitic drugs during pregnancy has remained largely empirical, with the notable exception of a recent study of antimalarial drugs in African women. Under Fansidar® treatment, when pregnant women were compared to women after delivery, there was an overall three-fold higher clearance for sulfadoxine [31–33], which is in agreement with our results for SA, although both drugs do not pharmacologically behave fully identically.

No prior research exists on the effect of long-term storage at ≤ − 18 °C on the stability of PY and SA, as was the case for the samples derived from patients with OT but not with PT. Some data suggest that PY may be stable for at least 91 days when stored at room temperature or at 4 °C and for several months at − 20 °C [34, 35]. In order to exclude a major impact of sample storage conditions and in the absence of published data on females under standard PY and SA therapy [18], we chose to include samples from a small group of males with OT where the same sampling, storage, and analysis protocols had been followed (Table 2). As the median plasma levels from these males (PY 1321 [962; 2140] µg/l; SA: 82.4 [53.5; 115.0]mg/l) are in good agreement with published values for males (PY 1887 ± 1161 µg/l; SA 42.26 ± 12.28 mg/l up to 84.9 ± 23.5 µg/ml) [15, 36], we have no evidence that freezing could have affected any of the measured concentrations in OT patients and that our findings are therefore reliable and robust.

## Conclusions

Our data indicate that insufficient drug levels for both drugs were found in every sixth patient with pregnancy-acquired toxoplasmosis, which could only partially be explained by the time interval between drug intake and blood sampling not being recorded, as well as missing information pertaining to the co-medication and pregnancy-associated pharmacologic changes. Observed median PY and SA concentrations in pregnant women were 34% below the concentrations seen in non-pregnant patients treated for active OT. Against the backdrop of a long controversy on the efficacy of prenatal *Toxoplasma* therapy with regard to clinical outcomes in newborns, we need to clarify how these concentrations can be explained and to what extend the observed lower-end ranges of plasma levels for PY and SA in pregnant women and a plasma concentration one-third of the maternal level in the foetus [35] may influence the efficacy of the drugs in the foetus and newborn in future studies. Systematic measurements of plasma drug concentrations are an important option to objectively control for compliance, as well as for other factors of influence and relevant for adopting the treatment regime [37]. Prospectively used, these may hold promise to close the gap between expected and observed outcomes of pregnancy in human PT.

## Data Availability

Data will be made available upon request to the corresponding author.
